# Enhancement of Activated Carbon on Anaerobic Fermentation of Heavy-Metal-Contaminated Plants: Insights into Microbial Responses

**DOI:** 10.3390/microorganisms12112131

**Published:** 2024-10-24

**Authors:** Yonglan Tian, Huayong Zhang, Lei Zheng, Yudong Cao, Wang Tian

**Affiliations:** Research Center for Engineering Ecology and Nonlinear Science, North China Electric Power University, Beijing 102206, China; rceens@ncepu.edu.cn (H.Z.); 15600462123@163.com (L.Z.); caoyd1995@hotmail.com (Y.C.); tianwang@ncepu.edu.cn (W.T.)

**Keywords:** heavy-metal-contaminated plants, anaerobic fermentation, activated carbon, microbial properties

## Abstract

Anaerobic fermentation is a potentially cost-effective approach to disposing of metal-contaminated biowaste collected during phytoremediation. However, the compound heavy metals contained in the biowaste may limit the efficiency of anaerobic fermentation. In this study, anaerobic fermentation with alfalfa harvested from an iron tailing as the feedstock was set up and further enhanced by granular activated carbon (AC). The results showed that adding AC improved the cumulative biogas yields of alfalfa contaminated with metals (AM) by 2.26 times. At the biogas peak stage, plenty of microbes were observed on the surface of the AC, and the functional groups of AC contributed to better electron transfer, lower heavy metal toxicity and higher CH_4_ contents. AC increased the richness and decreased the diversity of bacteria while reducing both the richness and diversity of archaea. The AC addition resulted in higher relative abundance of *Prevotella*_7, *Bacteroides* and *Ruminiclostridium*_1, which enhanced the hydrolysis of substrate and produced more precursors for methanogenesis. Meanwhile, the relative abundances of *Methanosarcina* and *Methanobacterium* were remarkably increased together with the metabolism of cofactors and vitamins, indicating the enhancement of both the acetoclastic and hydrotrophic methanogenesis. The present study provided new insights into the microbial responses of the anaerobic fermentation in heavy-metal-contaminated plants and proved the possibility of enhancing the biogas production by AC.

## 1. Introduction

Heavy-metal-polluted and metalloid-polluted soils have caused worldwide concern since a healthy soil environment is crucial for the food safety and living standards [1,2]. In particular, climate change is expected to cause the release of toxic metals, which calls for efficient and practical technology to help with soil remediation [3]. As one of the developing hotspots of soil remediation technology, phytoremediation shows great potential in remediating heavy metal contamination via the enrichment and hyperaccumulation of heavy metals by plants such as annual or semiannual crops [1,4]. The rapid growth of those plants produces a large amount of lignocellulosic biomass, which is typically incinerated and can also be used as a feedstock for bioenergy productions such as anaerobic fermentation [1,5]. During the process of anaerobic fermentation, many functional enzymes contain transition metals as cofactors for electron transport or as catalytic centers at active sites [6,7]. These metals released from the contaminated plants are of importance for fermentative microorganisms’ optimal growth and performance. More importantly, the fractionation of heavy metals, rather than the total concentrations or contents, is crucial for the anaerobic fermentation process [8,9]. Although many kinds of biomass-containing compounds of heavy metals have been proven to enhance biogas production to some extent [5,9,10], there was still the potential for further degrading of feedstocks and the production of more biogas from those contaminated biowastes.

The efforts regarding promoting biogas production from metal-contaminated biomass are focusing on alleviating the toxicity of over-released heavy metals and improving biodegradation. Specifically, activated carbon (AC) is currently most commonly used for adsorbing heavy metals and synchronously enhancing biogas production [8]. The addition of granular AC into the anaerobic digestion of different substrates (e.g., organic fraction of municipal solid waste, wheat husks, swine manure) has caused an improvement in biogas yields [11]. Granular AC provided microorganism attachment sites and functional groups for electron transfer, improving the hydrolysis of dry anaerobic digestion fed with pig manure and corn straw [12]. Biochar, as a precursor of AC, was also reported to be helpful in the formation and degradation of intermediate acids, leading to the enrichment of the methanogenic archaea, a reduction in the reaction time and an enhancement of the electron transfer and, finally, methane yield [13,14]. Moreover, it was able to improve the stability of the fermentation process by alleviating the inhibitory effects of excessive inhibitors (ammonia, heavy metals and so on) [14,15,16]. The presence of functional groups (such as –NO_2_, –Cl, –NH_2_, =CO and –COOH) at the surface also accounted for its suitability regarding mitigating the higher acidity of the anaerobic reactor [15]. Microbial attachment and acclimation, selective colonization of functional microbes, and promotion of syntrophic metabolisms have been identified as the plausible routes for promotion of biomethanation in the presence of biochar [17]. Lei et al. [18] incorporated granular AC into a bioreactor fed with raw incineration leachate and found that granular AC stimulated the growth of methanogens and insoluble-metal-reducing bacteria. In particular, granular AC enriched *Methanosarcina* and *Geobacter* species that can participate in direct interspecies electron transfer (DIET) and improve the performance of methanogenic bioreactors [18]. Although the impacts of AC additions have been validated by various researchers, a common mechanism has not been agreed upon. Most of all, none of the previous studies revealed the role of AC during the anaerobic fermentation fed with compound heavy-metal-contaminated plants in which heavy metals were released gradually and performed in various forms [9]. The asynchronism of AC adsorption and metal release resulted in different performances and mechanisms of the anaerobic fermentation process.

This research studied the effect of AC on the anaerobic fermentation of heavy-metal-polluted alfalfa. The biogas properties, AC properties, structure and functions of the bacterial and archaeal communities were investigated to reveal the role and mechanism of AC. The purpose of this study is to provide valuable information for mechanism research on enhancing the anaerobic fermentation of heavy-metal-polluted plants by introducing AC as well as promoting the biomethanation of metal-contaminated biowaste in practice.

## 2. Materials and Methods

### 2.1. Experimental Materials

The compound-metal-contaminated alfalfa (AM), *Medicago sativa* L., was collected from an abandoned iron mining area in Zhangjiakou City, Hebei Province, China, and the aboveground biomass was used as one of the feedstocks. The harvested alfalfa was air-dried until moisture levels reached <10%; it was then ground into powder and passed through a 10-mesh sieve. Fresh cow dung (CD) collected from a dairy farm in Zhangjiakou was stored at 4.0 °C and was used as both the inoculum and another feedstock. No extra inoculum was used to start the co-fermentation experiments. The properties of the alfalfa and the cow dung were the same as with the previous study [9]. Briefly, the total solids (TS) of the AM and CD were 95.63 ± 0.21 and 29.70 ± 0.66% dry weight, respectively. The volatile solid (VS), total nitrogen (TN), total organic carbon (TOC), cellulose, hemicellulose and lignin of the AM and CD were 89.07 ± 0.72 and 82.06 ± 0.58, 0.62 ± 0.10 and 0.25 ± 0.20, 23.64 ± 0.15 and 10.53 ± 0.00, 36.99 ± 0.27 and 21.64 ± 0.48, 24.74 ± 0.75 and 13.32 ± 0.88, 23.55 ± 0.15 and 16.22 ± 0.62%TS, respectively. The contents of Fe, Cu, Ni and Zn in the AM and CD were 2000.00 ± 82.12 and 610.80 ± 12.34, 13.25 ± 0.25 and 19.97 ± 0.30, 1.19 ± 0.37 and 10.00 ± 0.10, 42.00 ± 1.61 and 83.60 ± 1.59 μg/g, respectively. The contents of Cd and Co were negligible (lower than the limit of detection). The granular AC (Cas No.: 7440-44-0) was purchased from Shanghai Macklin Biochemical Technology Co., Ltd. (Shanghai, China). The micropore volume of the AC was 0.48 cm^2^/g. The BET and Langmuir specific surface areas were 660.8 ± 16.3 m^2^/g and 832.9 ± 5.5 m^2^/g, respectively.

### 2.2. Anaerobic Fermentation Experiment Set Up

Three experimental groups were set up: the CD group (with CD only as both feedstock and inoculum), the CD+AM group (mixed CD and AM as feedstocks) and the CD+AM+AC group (further adding 1 g granular AC into the CD+AM group). The volume of the Erlenmeyer flask used for the fermentation experiment was 500 mL, with a working volume of 300 mL. The TS concentration in the reactor was 8%. According to the previous study, the dry weight ratio of the alfalfa and cow dung for the CD+AM group was set as 1:2 (8 g alfalfa:16 g cow dung) [9]. The fermenters were purged with N_2_ for 1 min to remove the oxygen in the system before the beginning of the experiments. The temperature of the fermenter was controlled by a constant-temperature water bath (37.0 ± 1.0 °C). The experiments lasted for 31 days, and the substrates were mixed by shaking every morning between 8:30 AM and 9:00 AM prior to sampling and measurements.

### 2.3. Measurements

Biogas yields were measured daily after shaking by measuring the volume of the discharged saturated saline solution. The percentages of CH_4_ in the biogas were measured by a gas chromatograph (GC–2014C, Shimadzu Co., Kyoto, Japan), as previously reported [9].

At the beginning of the experiment, a total number of 30 Erlenmeyer flasks were prepared with the same feedstocks for each group. During the experiment, samples were collected from three of the Erlenmeyer flasks on the 7th, 10th, 13th, 16th and 19th day. After the experiment, the peak daily biogas yields were recorded on the 13th and 16th day for the CD+AM and CD+AM+AC groups, respectively. Then, samples collected on these two days were sent for metal concentrations and microbial community while granular AC was picked out for analysis of the characteristics. The scanning electron microscope (SEM) and Fourier transform infrared absorption spectroscopy (FTIR) of the AC was measured by Beijing Zhongkebaice Technology service Co., Ltd. (Beijing, China). The metal concentrations in the granular AC were determined by an inductive coupled plasma emission spectrometer (ICP-OES 730, Agilent Technologies Inc., Santa Clara, CA, USA) after being extracted by hydrochloric acid–nitric acid (1:3).

The measurements of the microbial communities in the samples collected at the peak stage (13th day for the CD+AM group and 16th day for the CD+AM+AC group) were carried out by the Beijing Allewgene Technology Co. Ltd. (Beijing, China) after certificating at 8000 rpm and 4 °C for 3 min. In brief, the genomic DNA of the samples was extracted using the E.Z.N.A. Soil DNA Kit (Omega Bio-tek, Inc., Norcross, GA, USA) following the manual. Concentration and quality of the genomic DNA were checked by a NanoDrop 2000 spectrophotometer (Thermo Scientific Inc., Waltham, MA, USA). The V3-V4 hypervariable region of bacterial 16S rRNA gene and archaeal 16S rDNA were amplified with primers 338F (5′-ACTCCTACGGGAGGCAGCAG-3′) for bacteria, 344F (5′-ACGGGGYGCAGCAGGCGCGA-3′) for archaea and 806R (5′-GGACTACHVGGGTWTCTAAT-3′) for both bacteria and archaea. Sequencing libraries were generated using a NEB Next Ultra II DNA Library Prep Kit (New England Biolabs, Inc., Ipswich, MA, USA) following the manufacturer’s recommendations. The constructed library was then qualified and PE250 was used for sequencing. After data splitting and splicing, Vsearch [19] (v2.7.1) software was used to remove sequences with lengths less than 230 bp, and the chimeric sequence was removed by the UCHIME method [20] according to the Gold Database (https://gold.jgi.doe.gov/). The run, image analysis, base calling and error estimation were performed using Illumina Analysis Pipeline Version 2.6 (Illumina, Inc., San Diego, CA, USA). Qualified sequences were clustered into operational taxonomic units (OTUs) at a similarity threshold of 97% using the Uparse [21] algorithm of the Vsearch (v2.7.1) software. The BLAST [22] tool (2.6.0+) was used to classify all OTU representative sequences into different taxonomic groups against the Silva138 Database [23] (https://www.arb-silva.de/) and the e-value threshold was set to 1 × 10^−5^. The microbial communities were then obtained after annotation.

### 2.4. Data Analysis

The data in the study were the average of three repeats. Error bars represent the standard errors of the mean: SEM = SD/$\sqrt{n}$, where SD is the standard deviation. An analysis of variance (ANOVA) was used for analyzing the differences in microbial diversity indices among groups. The diversity analysis and the Phylogenetic Investigation of Communities by Reconstruction of Unobserved States 2 (PICRUSt2) function prediction were completed using the Wekemo Bioincloud (https://www.bioincloud.tech) [24] accessed on 15 September 2024.

## 3. Results and Discussion

### 3.1. Enhancement of AC on the Biogas Production of Metal Contaminated Alfalfa

**Figure 1:**
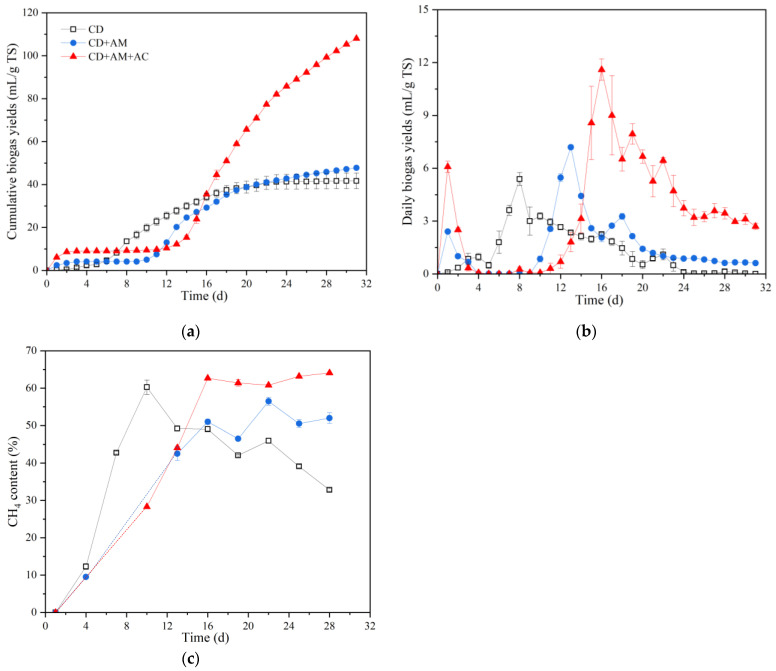
Cumulative biogas yields (**a**), daily biogas yields (**b**) and CH_4_ contents (**c**) of the CD, CD+AM and CD+AM+AC groups during the fermentation. CD: cow dung only; CD+AM: cow dung and alfalfa; CD+AM+AC: cow dung, alfalfa and activated carbon.

The cumulative biogas yields of the CD, CD+AM and CD+AM+AC groups are shown in Figure 1a. The CD+AM group produced more biogas than the CD group, while AC enhancement further improved the cumulative biogas yields by 2.26 times. It indicated that the over-loaded Fe in the alfalfa as well as its interactions with other metals limited the degradation and biogas production of alfalfa, which was indeed alleviated by introducing AC into the fermenter.

The release of heavy metals by alfalfa fermentation inhibited the daily biogas production at the beginning of anaerobic fermentation and resulted in the stagnation period in the CD+AM and CD+AM+AC groups (Figure 1b). The maximum daily biogas yield, 11.60 mL/g TS, was obtained in the CD+AM+AC group on the 16th day, which was 2.12 times higher than the peak daily biogas yield of the CD+AM group (obtained on the 13th day). Therefore, adding AC was able to improve but delay the daily biogas peak of the heavy-metal-polluted alfalfa anaerobic fermentation. The promotion of AC in the biogas production has been largely reported [14,18,25]. In contrast, a previous study suggested that biochar effectively shortens the lag phase and alleviates inhibitor stress during the anaerobic digestion process [14]. In this study, the lag phases of the CD+AM and CD+AM+AC groups could be attributed to the refractory lignocellulose of alfalfa and the release of heavy metals.

The CH_4_ contents of the CD+AM+AC group were, on average, higher than that of the CD and CD+AM groups (Figure 1c). Moreover, the CH_4_ content of the CD+AM+AC group was more stable after the daily biogas peak (16th day), with an average content of 62.42%. The promoting effect of AC on CH_4_ content was due to the positive role of AC in biogas upgradation, i.e., in situ and ex situ removal of the impurities present in biogas, viz., CO_2_ (25~30%), H_2_S (0~0.5% by volume), ammonia (0~5% by volume), water vapor (1~5%), along with some dust particles, nitrogen, and siloxane [26]. The biogas upgradation of AC was realized by its high porosity, large surface area of basic, hydrophobic sites and its specific functional groups [15]. For example, Shen et al. [27].reported a sequestration of CO_2_ (54.9~86.3%) and H_2_S (<5 ppb) using biochar during thermophilic anaerobic digestion of waste-activated sludge, increasing the CH_4_ concentration by up to 96.7%. Kanjanarong et al. [28] found that 98% H_2_S was removed during anaerobic treatment of sulfate-laden wastewater. In the present study, the CH_4_ contents of the AC-added group was increased but still in the normal range. It was speculated that elevating the amount of AC would further improve the CH_4_ proportion.

### 3.2. Properties of Activated Carbon

**Figure 2:**
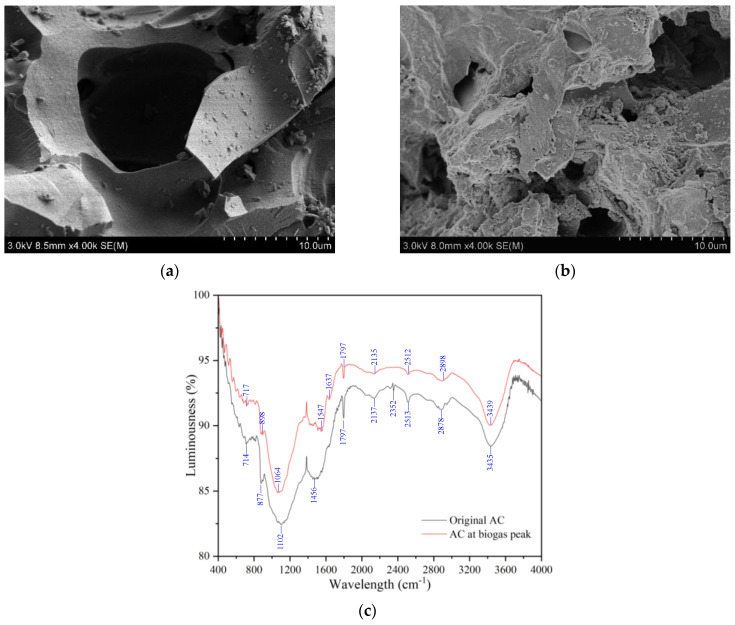
Scanning electron microscope (SEM) and Fourier transform infrared absorption spectros-copy (FTIR) of the AC. (**a**) SEM of the original AC; (**b**) SEM of AC collected on the daily biogas peak day; (**c**) FTIR of original and peak day AC.

In order to clarify the roles of AC in anaerobic fermentation, the SEM of AC on the daily biogas peak day (i.e., 16th day for the CD+AM+AC group) was determined and compared with the original AC. The surface of the original AC was generally smooth, with a few detrital particles of carbon or salt crystals (Figure 2a). After fermentation, the microorganisms grew and colonized on the surface of the AC and resulted in a rugged surface appearance. The closely attached biofilm on the surface of the AC was conducive to improving the DIET efficiency [29]. Moreover, the direct adsorption of heavy metals by AC and the indirect adsorption by the attached biofilm may realize the quick and long-term immobilization of heavy metals, thereby reducing their toxicity to microorganisms.

The presence of surface functional groups, free radicals and metals/metal oxides contributed to the redox properties of AC [30]. According to the FTIR results, there were several functional groups in the original AC (for instance, C–C stretching (735~878 cm^−1^), C–O stretching (951~1200 cm^−1^), CH_2_/CH_3_ sym (1104~1249 cm^−1^), C=C aromatic (1392~1728 cm^−1^), C–H bend or scissoring (1450~1470 cm^−1^), C=O (1630~1780 cm^−1^), C=O stretching (1785~1815 cm^−1^), carboxylic O-H (2500~3000 cm^−1^), C–H stretch (2850~2950 cm^−1^), and OH stretching (3200~3550 cm^−1^) groups, etc. [31,32]). At the daily biogas peak stage, the luminousness was generally higher the original AC. Meanwhile, some of these groups were weakened, for instance, the carboxylic O–H and C–H stretch groups. The shifts of some functional groups, such as C–O stretching, C=O and carboxylic O–H, indicated the adsorption of heavy metals [32,33]. This result coincided with the heavy metal contents determined in the original AC and the AC collected at the daily biogas peak (16th day). Fe, Cu, Ni, Zn were 1.1578 and 1.5964, 0.0027 and 0.0040, 0.0005 and 0.0029, 0.3255 and 0.0187 mg/g in the original AC and AC collected at the daily biogas peak, respectively. Therefore, Fe, Cu and Ni were adsorbed by AC, while Zn in the original AC was released into the fermenter (the reason for this requires further research). The adsorption of heavy metals by AC reduced their toxicity and alleviated their inhibitory effects on biogas production.

As previously reported, C=O groups were also responsible for the electron-donating capacity of AC [15]. The C=O and OH group contributed to the removal of H_2_S from the biogas [28]. Furthermore, the O-containing functional groups contained on the surface of biochar was positively correlated with the cationic exchange capacity (CEC) [13]. The lone pair electrons in the O atom of functional groups reduced the heavy metals’ biological activity by coordination bonds [34]. In other words, those O-containing functional groups were important in immobilizing heavy metals in the anaerobic systems, thereby reducing the inhibitory effect of heavy metals on microorganisms and biogas production. Therefore, the stimulatory effects of the AC functional groups contributed to better electron transfer, lower heavy metal toxicity, and higher CH_4_ proportions in the biogas.

### 3.3. Microbial Properties

#### 3.3.1. Diversity of Microbial Communities

**Figure 3:**
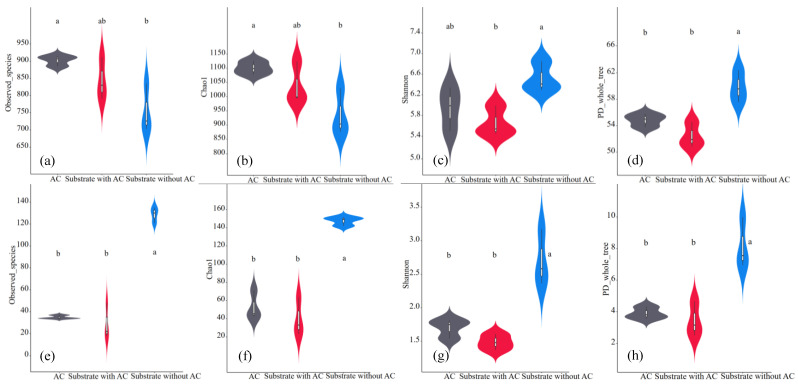
α-diversity indices of the microbial communities in the substrate without AC, the substrate with AC and on the surface of AC. (**a**,**e**) Observed species; (**b**,**f**) Chao 1 index; (**c**,**g**) Shannon index; (**d**,**h**) PD_whole_tree; (**a**–**d**) bacteria; (**e**–**h**) archaea. Different letters indicate the significant differences based on the ANOVA analysis.

The impacts of heavy metals on the microbial diversity was controversial due to the type, concentration and form of the heavy metals [35]. Normally, AC would increase the diversity of a bacterial community [29]. Nevertheless, the coupling effects of AC and heavy metals on the microbial diversity under anaerobic conditions were not previously reported. In the present study, the bacterial and archaeal α-diversity indices of the substrate without AC (collected in the CD+AM group on the 13th day), the substrate with AC (collected in the CD+AM+AC group on the 16th day by carefully picking out AC) and granular AC (collected in the CD+AM+AC group on the 16th day) were calculated and shown in Figure 3. Obviously, the α-diversity indices of the archaeal community were much lower than that of the bacterial community, which was due to the lower growth rate of archaea than bacteria [36].

The impacts of the AC addition on the α-diversity of bacteria and archaea were different. For bacterial diversity, the richness (observed species and Chao 1) and diversity (Shannon and PD_whole_tree) indices on the surface of granular AC were averagely higher than that in the substrate with AC, but not significantly so (Figure 3a–d). There were no significant differences in the richness indices (observed species and Chao 1) between the substrates with and without AC. However, the diversity indices (Shannon and PD_whole_tree) were higher in the substrate without AC (Figure 3c,d). The responses of the bacterial community involved the coupling results of both heavy metals released from the alfalfa and the addition of AC. As previously reported, high concentrations of heavy metals usually reduce the diversity of bacterial communities [37,38]. However, the presence of carbonaceous materials (such as AC) may result in various responses in terms of richness and diversity due to the complex anaerobic fermentation conditions, feedstocks, properties of additives and so on [39,40]. In this study, adding AC into the fermenter adsorbed the heavy metals released from alfalfa and benefited for the richness of the bacterial community while decreasing its diversity. It indicated that the bacterial community in the presence of AC was composed of more remarkably different species than the substrate without AC.

All four α-diversity indices of the archaeal community in the substate with AC were significantly lower than that in the absence of AC (Figure 3e–h, *p* < 0.01). Therefore, AC reduced both the richness and diversity of the archaea. The possible reason was that the over-loaded Fe, especially soluble Fe [9], and its combining effects with other heavy metals released from the alfalfa inhibited the growth of dominant microorganisms, allowing other microorganisms to compete in a better manner, resulting in an increase in species diversity [41]. The addition of AC immobilized those soluble metals and diminished the inhibitory effects, thereby resulting in the lower microbial diversity [41]. However, similar to the preceding research, a higher diversity index of archaea was not directly related to better anaerobic fermentation performances since one group of methanogenic bacteria can be replaced by another group [42]. It was therefore necessary to reveal the composition of the microbial community.

#### 3.3.2. Structure of Microbial Communities

**Figure 4:**
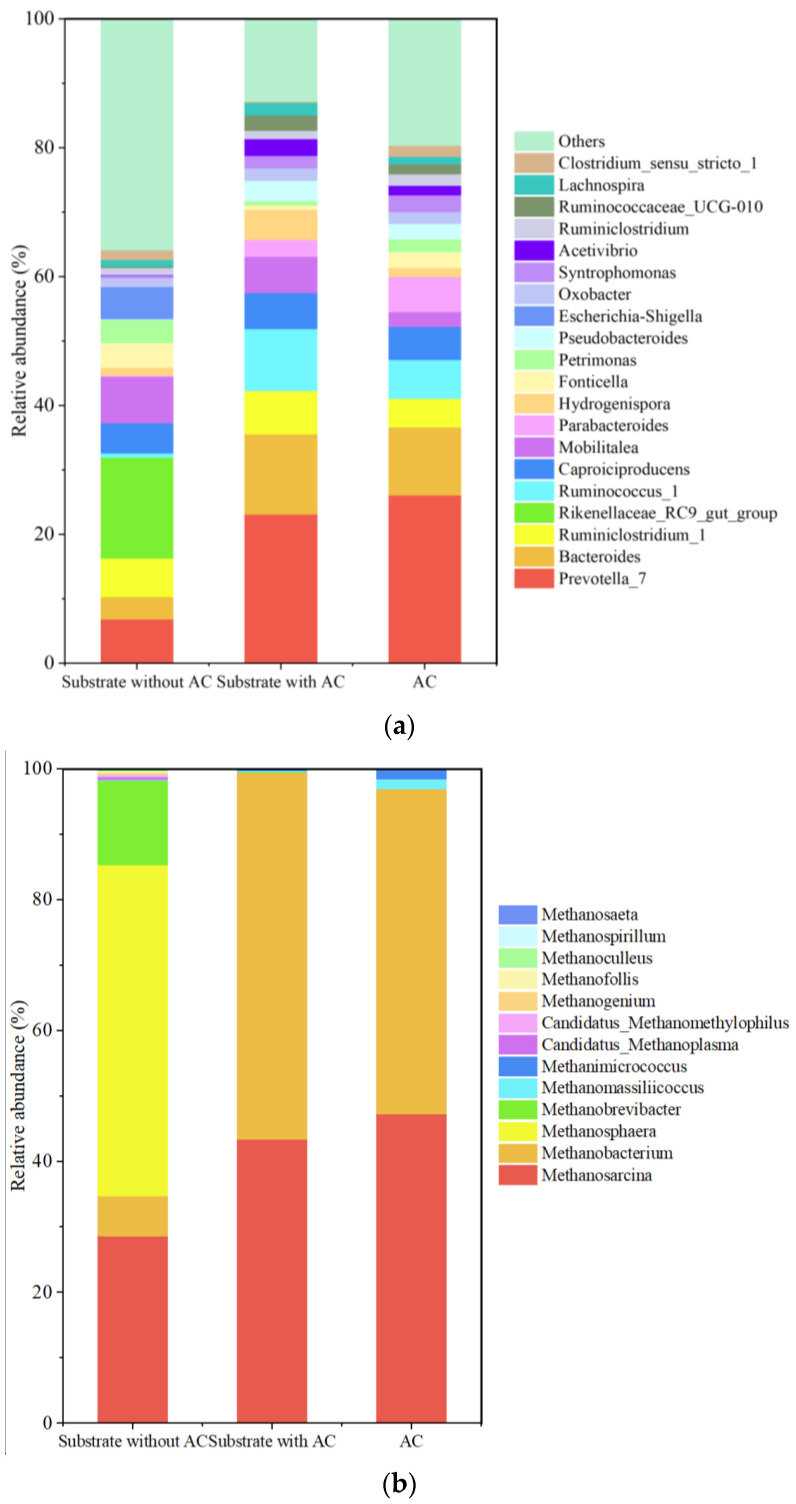
Bacterial (**a**) and methanogenic (**b**) genera in the substrate without AC, substrate with AC and on the surface of AC.

The variations in bacteria and methanogens annotated on the level of genus in the substrate without AC, the substrate with AC, as well as the genera colonized on the surface of AC are shown in Figure 4. The dominant bacterial genus in the substrate without AC was *Rikenellaceae_RC9_gut_group*, followed by *Prevotella_7*, *Mobilitalea*, *Ruminiclostridium_1*, and *Caproiciproducens* (Figure 4a). It indicated that carbohydrates and proteinaceous compounds in the feedstocks were efficiently fermented by *Rikenellaceae_RC9_gut_group* to produce acetic and propionic acid [43,44]. Meanwhile, cellulose was degraded by *Mobilitalea* and *Ruminiclostridium*, with the fermentation products being further decomposed into acetic acid, succinic acid, isobutyric acid, isovaleric acid and lactic acid by *Prevotella_7* [45]. After adding AC, the relative abundances of *Prevotella_7*, *Bacteroides* and *Ruminococcus_1* were greatly increased, while *Rikenellaceae_RC9_gut_group* was almost disappeared. At the same time, the relative abundances of *Ruminococcus_1* and *Caproiciproducens* were maintained. Therefore, the introduction of AC promoted the growth of *Ruminococcus*, which enhanced the degradation of cellulose [46], and the hydrolytic products, like glucose, were further decomposed by *Prevotella_7* and *Bacteroides* to produce intermediates such as volatile fatty acids (VFAs) and lactic acid, etc. [45]. The lactic acid was then metabolized by *Caproiciproducens*, with caproate (belonging to VFAs) as the product [46]. Those VFAs provided more precursors for the CH_4_ production.

As shown in Figure 4b, the dominate methanogens in the substrate without AC were *Methanosphaera* and *Methanosarcina*, suggesting the cooccurrence of methylotrophic, aceticlastic and even hydrogenotrophic methanogenesis [47,48] during the anaerobic fermentation of AM. After AC addition, the relative abundance of *Methanobacterium* was greatly increased, which manifested the enhancement of the hydrogenotrophic methanogenesis pathway [49]. *Methanobacterium* was reported to be involved in the expression of coenzyme F_420_ [49], which was reported to be enhanced by heavy metals like Cu, Fe, Zn and Ni [50]. These heavy metals were released from the alfalfa biomass during the fermentation experiments and thereby promoted the growth of *Methanobacterium*, the generation of coenzyme F_420_ and the enhancement of the hydrogenotrophic methanogenesis. Meanwhile, more *Methanosarcina* was detected, while *Methanosphaera* was negligible. The increment of *Methanosarcina* was coincident with the variation in the bacterial community in the presence of AC, as abovementioned, which produced higher VFAs (mainly composed of acetate acid) and favored the growth of *Methanosarcina* [51]. Previous studies showed that *Methanosarcina* species have large Ni- and Co-dependent proteomes (including Ni/Co transporters, Ni-dependent proteins, and B12-dependent proteins) and were able to reduce Fe^3+^ in nontronite and illite-smectite minerals using methanol and H_2_/CO_2_ as substrates [49]. More importantly, the DIET between *Methanosarcina* and *Geobacter* was enhanced by biochar, which acted as an electronic conductor to metabolize ethanol for growth of the syntrophic partners [14]. Furthermore, the syntrophy of hydrogenotrophic *Methanobacterium* and aceticlastic *Methanosaeta* stimulated the DIET while removing the high levels of acid and ammonium from the reactor [52,53]. Therefore, introducing AC into the anaerobic fermentation of metal-contaminated alfalfa stimulated both the acetoclastic and hydrogenotrophic methanogenesis pathways.

#### 3.3.3. Predicted Functions of Bacterial Communities

**Figure 5:**
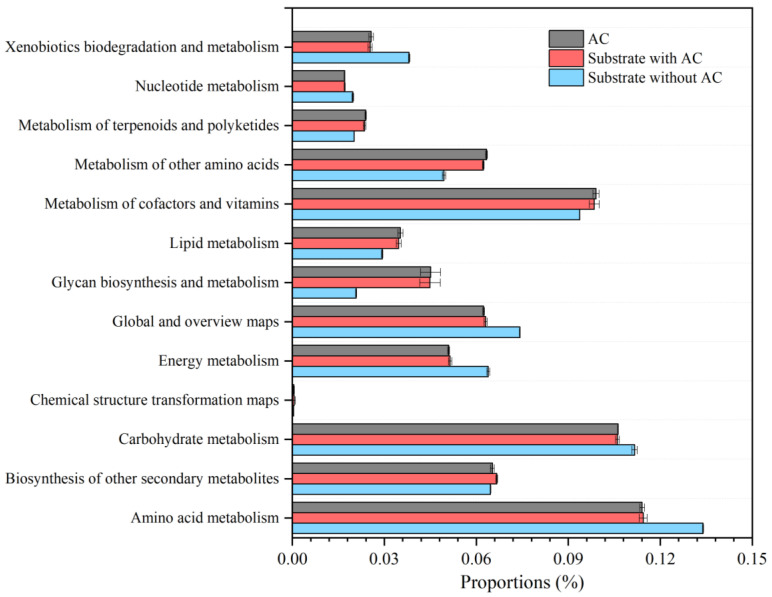
The KEGG level 2 functional pathways predicted by PICRUSt2.

To explore the impacts of adding AC on the functions of microbial communities, PICRUSt2 was used to predict the metabolic pathways using the Kyoto Encyclopedia of Genes and Genomes (KEGG) pathway metadata. The metabolic functions covered the pathways of (a) metabolism, (b) genetic information processing, (c) cellular processes, (d) environmental information processing, (e) human diseases, and (f) organismal systems. In the metabolism category, the most represented three KEGG pathways (level 2) were amino acid metabolism, carbohydrate metabolism and metabolism of cofactors and vitamins (Figure 5). Adding AC limited the metabolism of amino acids, carbohydrates, energy and xenobiotics. The reduction in amino acid metabolism was in line with the disappearance of *Rikenellaceae_RC9_gut_group*, which was responsible for the degradation of proteinaceous compounds [43,44]. The metabolism of carbohydrates was somehow compensated by glycan biosynthesis and metabolism. In contrast, the metabolism of cofactors, vitamins, glycans, lipids, terpenoids, polyketides and other amino acids were enhanced. The increased cofactor metabolism was in line with the abovementioned analysis on *Methanobacterium*, which was associated with the coenzyme F_420_ [49], manifesting the acceleration of AC on the hydrogenotrophic methanogenesis pathway.

## 4. Conclusions

The compound heavy metals accumulated in the biomass harvested from the contaminated land may limit the reutilization of those biowastes. This study proposed a new way to promote the biogas production of AM, i.e., alleviating the inhibitory effects of heavy metals by adding granular AC. The results suggested that using AM as feedstock was able to increase the biogas production of CD, which was further improved (by 2.26 times) by the addition of AC. The functional groups of granular AC alleviated the toxicity of over-loaded heavy metals and promoted biogas production. Adding AC enhanced the role of functional microorganisms that were capable of degrading the lignocellulosic compositions of the feedstocks. Meanwhile, the relative abundance of *Methanosarcina* and *Methanobacterium* were reinforced, resulting in the efficient acetoclastic and hydrogenotrophic methanogenesis pathways. The results of the present study provide references for future research on the compound-metal-stressed anaerobic fermentation process and reutilization of heavy-metal-contaminated biowaste.

## Data Availability

The raw data supporting the conclusions of this article will be made available by the authors on request.
